# DNA methylation profiles at hospital admission are associated with subsequent severe COVID-19 outcomes

**DOI:** 10.1186/s13148-026-02138-5

**Published:** 2026-04-22

**Authors:** Fei-Man Hsu, Harry Pickering, Steve E. Bosinger, Walter Eckalbar, Holden T. Maecker, Seunghee Kim-Schulze, Al Ozonoff, Joann Diray-Arce, Joanna M. Schaenman, Elaine F. Reed, Matteo Pellegrini

**Affiliations:** 1 Smart Medicine and Health Informatics Program, International College, National Taiwan University, Taipei, Taiwan; 2 Department of Molecular, Cell and Developmental Biology, University of California Los Angeles, Los Angeles, CA USA; 3 Department of Pathology and Laboratory Medicine, David Geffen School of Medicine, University of California Los Angeles, Los Angeles, CA USA; 4 Emory School of Medicine, Atlanta, GA USA; 5 Department of Medicine, University of California San Francisco, San Francisco, CA USA; 6 Stanford University School of Medicine, Palo Alto, CA USA; 7 Icahn School of Medicine at Mount Sinai, New York, NY USA; 8 Clinical and Data Coordinating Center, Boston Children’s Hospital, Boston, MA USA; 9 Division of Infectious Diseases, Department of Medicine, David Geffen School of Medicine, University of California Los Angeles, Los Angeles, CA USA

**Keywords:** COVID-19, DNA methylation, Epigenetics, SARS-CoV-2, Biomarker

## Abstract

**Background:**

SARS-CoV-2 infection leads to a wide range of clinical manifestations ranging from asymptomatic to fatal cases. DNA methylation plays a crucial role in modulating host responses to viral infections; however, its potential for forecasting COVID-19 disease progression has not been fully explored.

**Results:**

We aimed to explore the connection between DNA methylation and COVID-19 phenotypic trajectories by examining a subset of the IMPACC cohort (n = 75), which includes longitudinal samples from hospitalized COVID-19 patients grouped into five disease trajectory groups (TGs) based on respiratory severity during the acute phase of infection. Our findings reveal that DNA methylation is associated with respiratory status, hospitalization duration, and immune cell composition, including T cell depletion and band neutrophil accumulation during acute infection. DNA methylation profiles at hospital admission differ among TGs and are associated with subsequent disease progression. Furthermore, DNA de-methylation of specific distal enhancers correlated with TG, and DNA methylation patterns generally normalize post-resolution. Comparative analyses revealed that DNA methylation changes are more strongly associated with TGs than transcriptome profiles across time.

**Conclusions:**

This study highlights the association between DNA methylation and COVID-19 phenotypic trajectories and identifies CpG *loci* that could serve as potential biomarkers for identifying patients at heightened risk of severe COVID.

**Supplementary Information:**

The online version contains supplementary material available at 10.1186/s13148-026-02138-5.

## Background

The global pandemic of coronavirus disease 2019 (COVID-19) was caused by the RNA virus severe acute respiratory syndrome coronavirus 2 (SARS-CoV-2). The clinical manifestations of COVID-19 are highly variable, ranging from asymptomatic to fatal [1–3]. Previous research indicates that the severity predominantly depends on the host’s characteristics, e.g. age, comorbidities, ethnicity [1, 4, 5], and vaccine status [6].

DNA methylation patterns can change in response to host–pathogen interactions [7, 8]. It was previously reported that RNA viruses are capable of hijacking host immune cell epigenomes and inducing immune dysfunction [9], and several studies have characterized the epigenome perturbations in mild/asymptomatic [10] and severe COVID [11, 12] compared to non-COVID controls. In mild/asymptomatic COVID-19 cases, some differentially methylated regions (DMRs) persist before, during and after SARS-CoV-2 infection with the patterns resembling autoimmune or inflammatory diseases, and it is also possible to distinguish patients’ stage of infection by their DNA methylome [10]. Compared to healthy individuals or other viral infection such as influenza and HIV, severe COVID-19 cases have unusually high neutrophil proportion [12]. In addition to immune system dysregulation, chronological age is one of the major risk factors for severe COVID-19 and death regardless of other age-related comorbidities. However, most COVID-19 DNA methylation studies dichotomize the cohorts to compare COVID over non-COVID, or trichotomize subjects into healthy, asymptomatic/mild and severe COVID, and the variations within groups are typically not considered.

The IMPACC (Immunophenotyping Assessment in a COVID-19 Cohort) study analyzed participant characteristics and captured longitudinal clinical course dynamics which led to the definition of five disease trajectories (TG) [1, 2, 13]. In this study, we profiled peripheral blood mononuclear cell (PBMC) DNA methylation with targeted bisulfite sequencing (TBS-seq) in a subset of 75 IMPACC patients recruited at the Ronald Reagan UCLA Medical Center to characterize the longitudinal epigenomic changes of the five disease trajectories. The probe panel captures genomics regions associated with immune responses and can be used to deconvolute cell types comprising PBMC. We estimated each cell type’s abundance across a 1-year window and identified differences among patient groups. The DNA methylation 1–2 days post admission profiles are distinct between TG and are associated with COVID-19 progression after hospitalization. While accounting for several covariates such as age, sex and cell type composition, we identified CpG *loci* that have a significant association with TG-specific epigenomic modulation. We also compared the IMPACC TBS-seq with an end-stage renal disease (ESRD) cohort which serves as the COVID-negative reference profiled with the same TBS-seq panel and determined that convalescent, post-COVID patients’ DNA methylome resets to a pre-COVID state. Lastly, we found that transcriptomic profiles are more associated with TG cross-sectionally but not longitudinally. Our results demonstrate that DNA methylation informs patients’ future outcomes.

## Methods

### IMPACC cohort characteristics

This study includes the IMPACC cohort enrolled between May 2020 and February 2021 in Ronald Reagan UCLA Medical Center, California, the United States. D614G-containing SARS-CoV-2 lineages rapidly became dominant across North America in mid-2020 [14, 15], followed by an increase in circulation of the Alpha (B.1.1.7) variant by early 2021 [16]. Eligible participants were hospitalized with symptoms or signs consistent with COVID-19 and had SARS-CoV-2 infection confirmed by polymerase chain reaction (PCR) [13]. The detailed study design, schedule for clinical data, biological sample collection and demographic information about the participants were previously described [1, 13]. Briefly, detailed clinical assessments and nasal, blood, and endotracheal aspirates (intubated participants only) were collected within 72 h of hospitalization and on days 4, 7, 14, 21, 28 after hospital admission. If a participant required escalation of care or was re-admitted to the hospital prior to day 28, additional samples were collected within 24 and 96 h of care escalation or readmission. If participants were discharged prior to day 14 or 28, attempts were made to collect limited clinical information and biologic samples on days 14 and/or 28 in outpatients. Disease severity was assessed using a 7-point ordinal scale based on degree of respiratory illness,10 modified from Beigel et al. [17]. Specifically, it was assessed using the ordinal scale (OS) adapted from the World Health Organization COVID-19, and NIAID disease ordinal severity scales (OS1 = Not hospitalized, no limitations; OS2 = Not hospitalized, activity limitations, or requires home oxygen (O_2_); OS3 = Hospitalized, not requiring supplemental O_2_; OS4 = Hospitalized, requiring O_2_; OS5 = Hospitalized on non-invasive ventilation, or high-flow O_2_; OS6 = Hospitalized on invasive mechanical ventilation, and/or extracorporeal membrane oxygenation (ECMO); OS7 = Death) [1].

### Blood samples

8 ml of blood was drawn into the ACD tube. After Ficol density gradient centrifugation, PBMCs were separated isolated and cryopreserved in FCS/DMSO.

### Targeted bisulfite sequencing (TBS-seq)

#### Probe design

The probe panel design is based on the criteria to include CpG *loci* that (1) covers DNA methylation clock age estimators [18, 19], (2) has cell-type specificity, and 3) locates in the promoter regions (−1000 to + 250 bp from TSS) of viral response genes [20]. Biotinylated probes covering the selected CpG *loci* were synthesized by IDT (Integrated DNA Technologies). The coordinates of the targeted regions (GRCh38) are listed in Additional file 1: Table S1.

#### Library preparation

Genomic DNA was extracted from PBMCs using phenol–chloroform method [21]. 500 ng genomic DNA was sheared and subject to end-repair, A-tailing and ligated with methylated adaptors. Purified libraries were hybridized to biotinylated probes and subjected for bisulfite conversion (Zymo Cat# D5030). Captured DNA was PCR amplified with KAPA HiFi HotStart Uracil^+^ (Cat# KK2801) into a final TBS-seq library. Library quality was evaluated using TapeStation with the high-sensitivity D1000 tape (Agilent Cat# 5067-5584). A comprehensive TBS-seq protocol is demonstrated in [20]. The 182 TBS-seq libraries were randomized in 2 sequencing runs.

#### TBS-seq data processing

Illumina adapter sequences were trimmed off from the raw reads using Cutadapt [22] and only reads with minimum 30 bp were kept for downstream analysis. Trimmed reads were aligned to GRCh38 reference genome using *bsbolt align* function and the duplicated reads were marked with samtools markdup function before calling methylation using *bsbolt callmethylation* function [23]. CGmaps from all samples were aggregated into one methylation matrix using *bsbolt aggregatematrix* function with parameters -min-coverage 10 -min-sample 1.0.

#### Cell type deconvolution

A reference-based cell type deconvolution approach was used to estimate cell type composition with DNA methylation profiles [24]. To recapitulate cell type composition of PBMC, WGBS dataset from 6 cell types: B cell, CD4 T cell, CD8 T cell, NK cell, naïve T cell and monocyte were acquired from GSE186458 [25], and neutrophil band cell’s methylation profiles were from the Blueprint database [26]. In total, 34 WGBS profiles from each cell type with replicates were included (Additional file 1: Table S2). Cell type-specific differentially methylated regions (DMRs) were identified by one-versus-all comparisons using metilene [27] with the criteria to find DMRs that are (1) at least 500 bp, (2) with the delta methylation level < − 30%, and (3) with a false discovery rate (FDR) < 0.05. Cell type-specific CpG sites were further subtracted from each TBS-seq sample with *bedtools intersect* function and used as input files to deconvolute. A non-negative least square approach was applied to every TBS-seq profile and regress to the WGBS references for coefficient estimation. The detailed deconvolution data could be found in Additional file 2: Figure S3.

### RNA-seq

#### Library preparation

2.5 × 10^5^ PBMCs were lysed in 200uL Buffer RLT (Qiagen) and RNA was extracted using the Quick RNA MagBead Kit (Zymo) after DNase digestion. RNA quality was accessed using Qubit HS RNA assay (Thermo) and TapeStation (Agilent). cDNA synthesis was carried out with SMART-Seq v4 Ultra Low Input RNA Kit (Takara Bio) followed by the tagmentation, amplification and dual-indexing the library with the NexteraXT DNA Library Preparation Kit (illumina).

#### Transcriptome data pre-processing

IMPACC universal processing and quality control was performed using an internal Snakemake workflow for RNA-seq analysis (Github: https://github.com/yerkes-gencore/IMPACC-RNA_Seq). Reads were trimmed for adapter sequence and quality score with Cutadapt v1.14112 [22]. Reads were aligned with STAR v2.4.2a [28] to a composite reference of human (GRCh38) reference sequence with gene annotations from Ensembl (release 91) and SARS-CoV-2 (NCBI strain MN908947.3 [29]). Transcript abundance estimates were calculated internal to the STAR aligner using the algorithm of htseq-count [30].

### Blood CyTOF

The blood CyTOF workflow has been described before [2, 13]. In brief, several considerations have been applied to reduce sample usage, streamline sample processing, and minimize experimental variability [31]. Whole-blood samples are stained using a commercial lyophilized 30-marker panel designed to identify all major circulating immune cell subsets (Fluidigm Maxpar Direct Immune Profiling Assay), fixed and cryopreserved at the site of collection. The cryopreserved samples are shipped to the IMPACC co-Core Labs at ISMMS and Stanford for barcoded batched processing, where they are labeled with a supplemental panel of 14 additional antibodies targeting fixation-resistant epitopes to resolve additional dynamic changes in cell phenotype. To maximize reproducibility, the supplemental panel has been formulated as a cocktail and frozen in single-use aliquots for each processing batch. The labeled barcoded samples are frozen for batched acquisition. The resulting FCS files are evaluated using a centralized data processing pipeline including bead-based sample QC, data normalization and automated sample demultiplexing.

### Multivariate multiple linear regression (MMLR)

Per individual $i$, the methylation status of targeted locus (TBS-seq) or expression level of a gene (RNA-seq) $j$ is denoted as ${M}_{ij}$. Suppose every ${M}_{ij}$ is described by $k$ associated traits, i.e. multivariate, of each participant ${P}_{ik}$, that are weighted by per site coefficient ${C}_{kj}$, the methylation model is formulated as $Equation 1$,

1 $$M_{{ij}} = P_{{ik}} \times C_{{kj}} \left\{ {\begin{array}{*{20}l} {i\; \in \;number\;of\;recruits} \hfill \\ {j\; \in \;number\;of\;CpG\;loci} \hfill \\ {k\; \in \;number\;of\;traits} \hfill \\ \end{array} } \right. $$

This model represents a system of equations in which ${P}_{ik}$ and ${M}_{ij}$ are known variables. Our goal is to derive critical CpG sites for each trait, i.e. the unknown ${C}_{kj}$ that represents characteristics of sites, and could be achieved by solving Eq. 1 as,

2 $$ C_{kj} = P_{ik}^{\dag } \times M_{ij} $$

Here, $P_{ik}^{\dag }$ is derived through Moore–Penrose pseudoinverse of $T_{ik}$.

### Leave-one-out cross validation (LOOCV)

To avoid over-fitting, for each biological sample, a separate MMLR model was trained with the rest samples to derive $C$, and the trait prediction is made as,

3 $$ P_{ik} = M_{ij} \times C_{kj}^{\dag } $$

Here, $C_{kj}^{\dag }$ is the Moore–Penrose pseudoinverse of $C_{kj}$.

### Epi scores

For each trait $k$, the individual $i$ will have a predictive value ${P}_{ik}$ which we term an epi score. For instance, if $$k=\left\{\begin{array}{l}0 \;\left(LowTG\right) \\ 1 \;(HighTG)\end{array}\right.$$, the predicted value ${P}_{ik}$ is the epi trajectory score of this patient. To be noted, the prediction is independent of the observation, e.g. if the patients are not included in this cohort, once their DNA methylation profile is established, their epi scores could still be acquired by the MMLR model trained with this IMPACC cohort.

### Identification of trajectory-associated CpG sites

To identify statistically significant associations between low- and high-TG and the methylation per site, for each locus we estimated the significance of the coefficients described below,

4 $${y}_{m}={\beta }_{0}+{\beta }_{1}{x}_{m1}+{\beta }_{2}{x}_{m2}+\dots +{\beta }_{n}{x}_{mn}+\varepsilon $$

Here, ${y}_{m}$ is the methylation level at locus $m$, ${x}_{n}$ are the explanatory variables including age, sex, TG and cell type PCs. ${\beta }_{0}$ is the y-intercept, ${\beta }_{n}$ is the coefficient for each explanatory variable, and $\varepsilon$ is the error. For each CpG site ${y}_{m}$, $p$ values from the model per explanatory variable ${x}_{n}$ were derived and adjusted for multiple hypothesis testing with Benjamini–Hochberg correction. CpG sites with adjusted $p$ value < 0.05 regarding TG as $x$ were determined as TG associated sites.

### Functional enrichment analysis

Site-level GO enrichment analysis was performed using GREAT [32] with TG associated sites’ coordinates as foreground and all TBS-seq captured CpG sites as background. Gene-level GO enrichment analysis was conducted with Enrichr [33] and DAVID [34].

### Differential gene expression analysis

Gene counts from htseq-count were normalized using DEseq2 [35]. Differentially expressed genes were characterized between low- and high-TG with fold change > 2 and FDR < 0.05.

## Results

### Epigenomic profiling of participants within distinct clinical trajectory groups

We applied TBS-seq to a subset of 75 patients in the IMPACC cohort recruited between May 2020 and February 2021 at the Ronald Reagan UCLA Medical Center, California, the United States. The RNA-seq data of the same patients have been published previously [2]. Participant characteristics are listed in Table 1 and sample characteristics are shown in Additional file 1: Table S3. Five TGs were defined previously [1] using respiratory ordinal scores modeled across longitudinal observations of the entire IMPACC cohort (1164 participants) that represent the degree of respiratory support required, activity limitations and oxygen requirement [17] during the acute phase of SARS-CoV-2 infection (the first 28 days). TG1 had a short hospital stay (median[IQR] 3[2] days) and no limitations at discharge; TG2 required more respiratory support and longer stay (median[IQR] 7[4] days) than TG1 and no limitations at discharge; TG3 had similar respiratory support and hospital stay as TG2 (median[IQR] 7[7] days) but had limitations at discharge; TG4 required more aggressive respiratory support and had a prolonged hospital stay (median[IQR] 20[12] days) and TG5 needed high respiratory support and had fatal illness by day 28.

**Table 1:** Participant characteristics

	Participant	TG1	TG2	TG3	Low-TG	TG4	TG5	High-TG
Total n	75	9	13	15	37	31	7	38
Age median [IQR]	61 [52, 67]	54 [46, 62]	50 [41, 58]	65 [59, 68]	58 [44, 66]	63 [56, 67]	59 [56, 70]	63 [55, 69]
Sex n (%)								
Female	27 (36)	2 (22)	4 (31)	4 (27)	10 (27)	16 (52)	1 (14)	17 (45)
Male	48 (64)	7 (78)	9 (69)	11 (73)	27 (73)	15 (48)	6 (86)	21 (55)
Race n (%)								
Asian	4 (5)	2 (22)	1 (8)	1 (6)	4 (11)	0 (0)	0 (0)	0 (0)
Black	4 (5)	0 (0)	3 (23)	0 (0)	3 (8)	1 (3)	0 (0)	1 (3)
White	25 (34)	4 (45)	1 (8)	4 (27)	9 (24)	14 (45)	2 (29)	16 (42)
Other/declined	42 (56)	3 (33)	8 (61)	10 (67)	21 (57)	16 (52)	5 (71)	21 (55)
Ethnicity n (%)								
Hispanic	48 (64)	4 (44)	6 (46)	8 (53)	18 (49)	24 (77)	6 (86)	30 (79)
Non-Hispanic	27 (36)	5 (56)	7 (54)	7 (47)	19 (51)	7 (23)	1 (14)	8 (21)

**Fig. 1:**
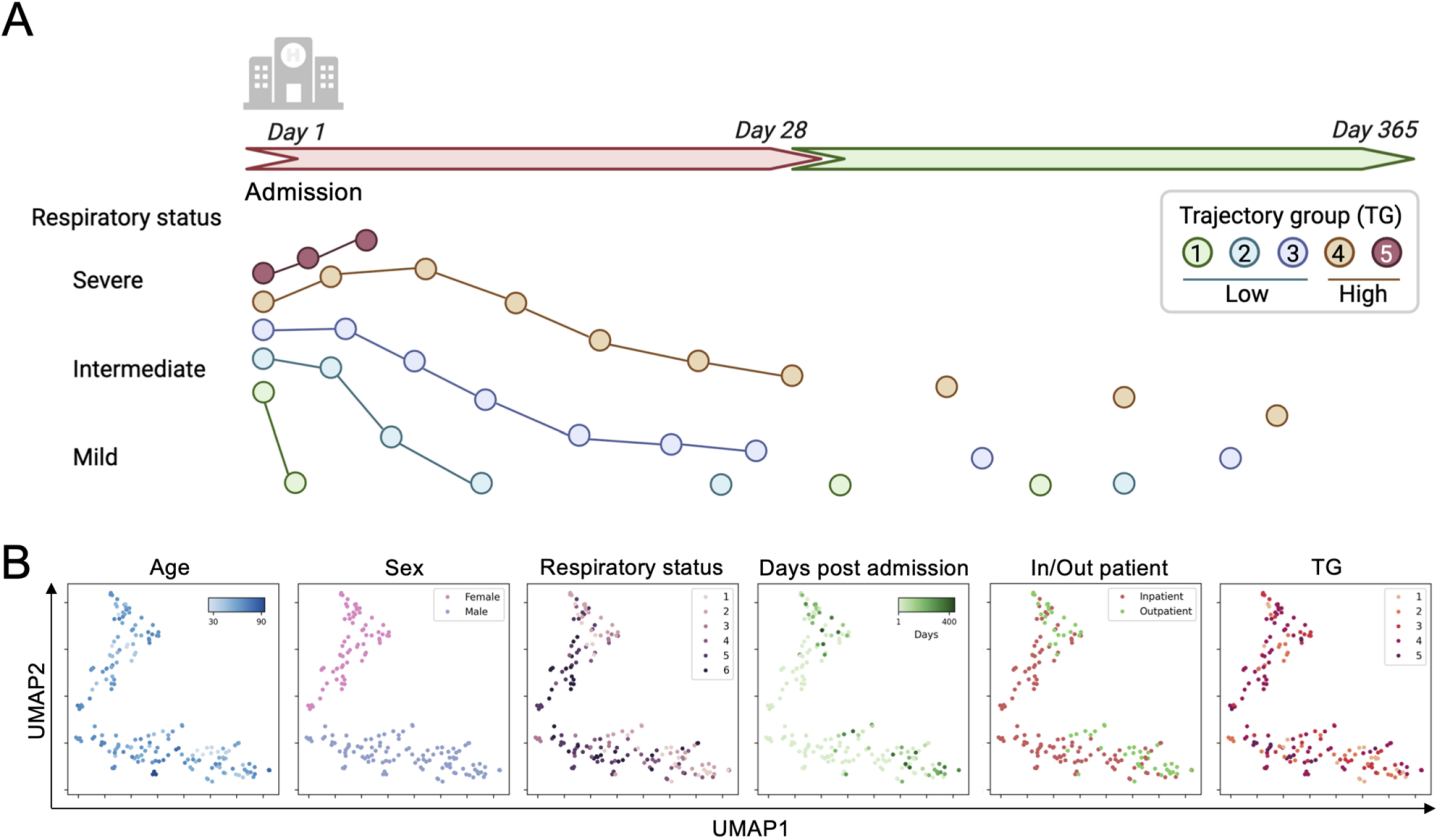
Longitudinal DNA methylation change across COVID-19 progression. **a** Study overview. PBMC samples were collected at different days post admission and profiled with TBS-seq. Patients’ longitudinal respiratory status was recorded, and the TG were determined by longitudinal clustering. **b** DNA methylome visualized with traits in UMAP coordinates (n = 182). Sex is the dominating trait to cluster DNA methylomes (Female n = 72, Male n = 110) and the DNA methylomes are further grouped by respiratory status and hospital stay

The study design is shown in Fig. 1a. Within a 1-year follow-up window, each patient was sampled multiple times post hospitalization, and the peripheral blood mononuclear cells (PBMCs) were isolated for TBS-seq and RNA-seq. Figure S1A shows every collection timepoint per individual in this study. The TBS-seq panel design is described in “Methods”, and the probes are enriched for genomic regions related to virus defense (Additional file 2: Fig. S1). We confirmed the capture rate was > 75% for all samples and 42,271 CpG *loci* across 182 samples with a sequencing depth ≥ 10X used for downstream analysis.

The Uniform Manifold Approximation and Projection (UMAP) dimensionality reduction shows that the DNA methylomes are associated with respiratory status and days post admission (Fig. 1b) in addition to sex which is one of the main factors that affects DNA methylation [36]. Moderate association with TG is also observed (Fig. 1b). These 3 traits are correlated with principal component 1 (PC1) that explained the most variance (25.8%) in the DNA methylome (Additional file 2: Fig. S2). The sex-stratified UMAPs further strengthen these associations are observed in both sexes (Additional file 2: Fig. S2). These results suggest there are longitudinal changes in DNA methylation that result from severe COVID-19.

### Blood cell composition is altered by SARS-CoV-2 infection

**Fig. 2:**
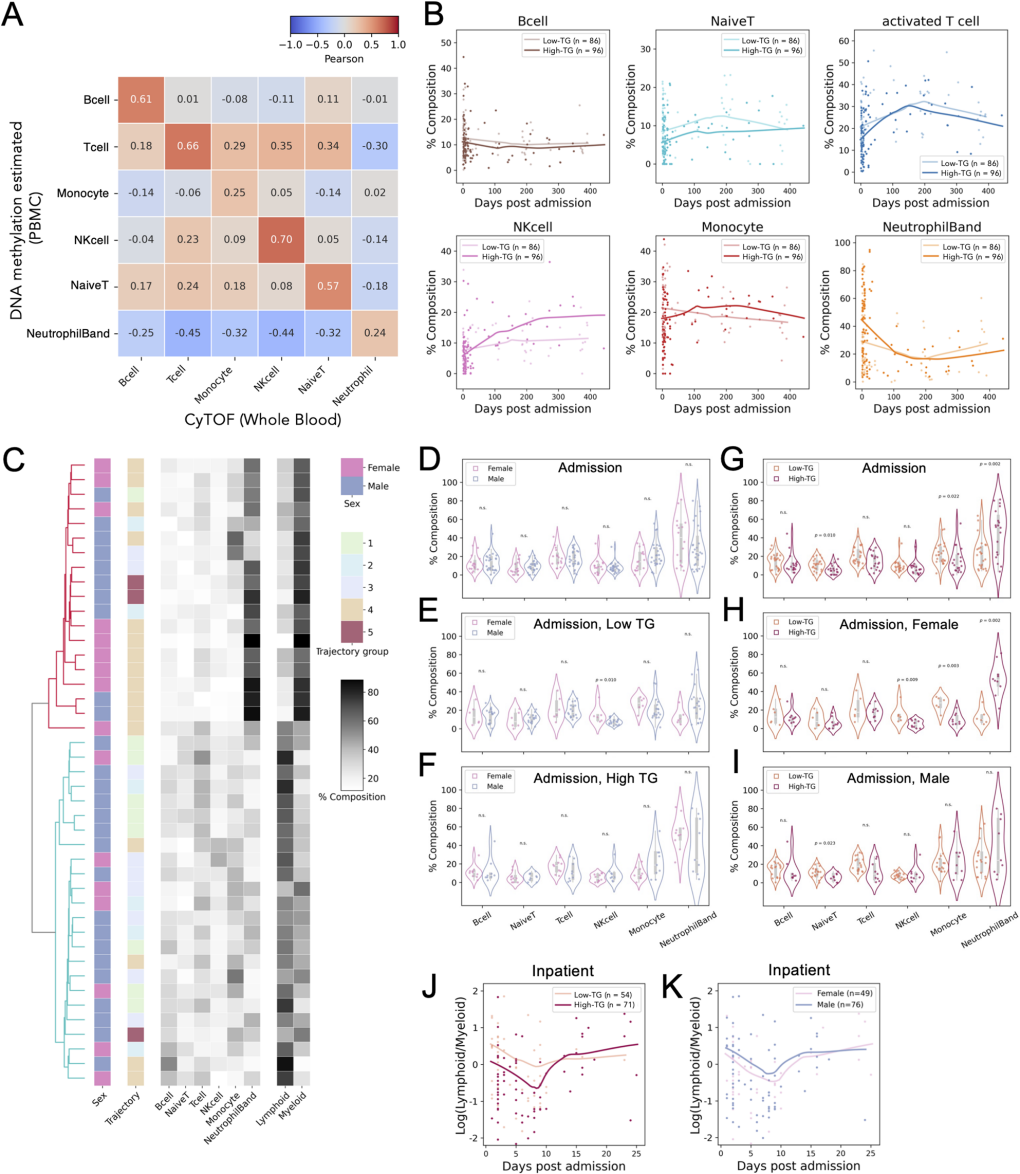
Leukocyte composition dynamics through severe COVID-19 hospitalization. **a** Cell type composition estimated with DNA methylation is correlated with the major cell type proportions as measured by whole blood CyTOF. **b** Longitudinal differences between high- (n=96) and low-TG (n=86) of each cell type’s composition. **c** Unsupervised hierarchical clustering of admission samples (n=43) shows cell type composition stratifies the DNA methylomes. **d** Admission leukocyte composition shows no difference between sexes but **e** female patients (n=16) possess more NK cell in low-TG and **f** the neutrophil band ratio surges homogeneously in high-TG female patients (n=10). **g** Admission leukocyte composition shows difference between TGs and **h** only female patients (n=16) possess excessive neutrophil band but **i** not male patients (n=27). **j** Inpatient lymphocyte/myelocyte ratios of high- (n=71) and low-TG (n=54). **k** Inpatient lymphocyte/myelocyte ratios of male (n=76) and female (n=49)

PBMC contain different leukocytes, and the composition significantly impacts DNA methylation. We performed deconvolution using whole genome bisulfite sequencing (WGBS) references (see “Methods”) to estimate the abundance of six cell types*;* B cells, naïve T cells, T cells, nature killer cells (NK cells), monocytes and band neutrophils, (Fig. 2 and Additional file 2: Fig. S3). Paired CyTOF data was acquired from whole blood as described previously [2], and a positive correlation was observed between the cell composition estimated from DNA methylation and CyTOF-determined frequencies in all cell types, especially lymphoid cells (Fig. 2a). Monocytes and neutrophils, i.e. myeloid cells, show weaker correlation (Pearsons’ r = 0.25 and 0.24). This is expected as PBMC samples have different myeloid cell composition compared to whole blood in which the monocyte signals could be confounded by the presence of granulocytes whereas mature granulocytes are removed in PBMC through gradient centrifugation.

To compare the cell composition dynamics during the course of COVID-19, we grouped TG1-3 as low-TG (n = 86) and TG4-5 as high-TG (n = 96) and displayed DNA methylation-based cell composition estimates across a 1-year follow-up window with trend lines determined using locally weighted scatter plot smoothing. We observed a clear difference between the trends of adaptive and innate immune cells, with high-TG patients having less B and T cells (Fig. 2b). NK cells and monocytes have similar trends with high-TG patients having a lower percentage at the day of admission, surpassing the low-TG group after hospitalization, and converging longitudinally. Most severe COVID-19 patients have high percentages of band neutrophils at admission, as much as 40% of PBMC in some high-TG patients at the day of admission (Fig. 2b). The high fraction of band neutrophils typically drops after hospitalization.

Next, we asked whether cell type composition is associated with disease severity at admission. Here we included samples from day 1–2 post admission (n = 43) as the “admission”. Among all admission samples, respiratory status 3–4 and 5–6 are dichotomized as intermediate and severe. NK cell (p = 0.025), naïve T cell (p = 0.002) and non-naïve T cell (p = 0.014) percentages were lower in severe cases with higher percentages of band neutrophils (Fig. 2c). We further grouped the immune cell types based on their hematopoietic lineage into myeloid and lymphoid cells and the composition differences between intermediate and severe cases are significantly different and in opposing directions (Fig. 2c). Due to the known sex differences in DNA methylation and potential differences in COVID-19 outcome by sex, we further split the analysis by sex and found the high fractions of band neutrophil cells were more significant in female than male between low- and high-TG (Fig. 2d–f), indicating a potential sex-specific immune response. When we stratify admission samples by TG, the high band neutrophil fractions are only observed in females (p < 0.01) (Figs. 2g-i). Throughout hospitalization, we found both TG (Fig. 2j) and sex (Fig. 2k) stratifies the lymphoid/myeloid ratio, specifically around 14 days post admission, with a lower ratio in high-TG and females. Taken together, our cell type estimation results are in line with previous studies that find that lymphopenia and emergency granulopoiesis are common features of severe COVID-19 and lead to altered immune responses [11, 12], and that high-TG and female sex both associate with lymphopenia.

### DNA methylation models are associated with days post admission and severity

To evaluate whether this TBS-seq probe panel has informative or diagnostic power, we built penalized regression models with days post admission as the response and the CpG methylation levels as the explanatory variables. We saw a moderate predict-actual correlation in the linear model (Pearson’s R = 0.52, Additional file 2: Fig. S5). When dichotomizing samples as early and late around day 14, 21 or 28, we obtained similar area under curve (AUC) values around 0.80 in binary classification (Additional file 2: Fig. S5), suggesting there is a significant change in DNA methylation from the acute phase of infection to longer-term post-hospitalization and the convalescent phase.

We next performed multi-class classification with respiratory status as the response variable (Additional file 2: Fig. S5). While the model can distinguish mild cases from more severe disease (AUC = 0.86) and shows a good performance to detect the severe cases from more mild disease (AUC = 0.77), it is unable to reliably differentiate the intermediate group (AUC = 0.56). Next, we dichotomized respiratory status 1–3 as moderate and 4–6 as severe, for which the binary classification yields an AUC of 0.79. This result suggests that moderate and severe are characterized by distinct epigenomes.

### DNA methylation at hospital admission is associated with TG after hospitalization

**Fig. 3:**
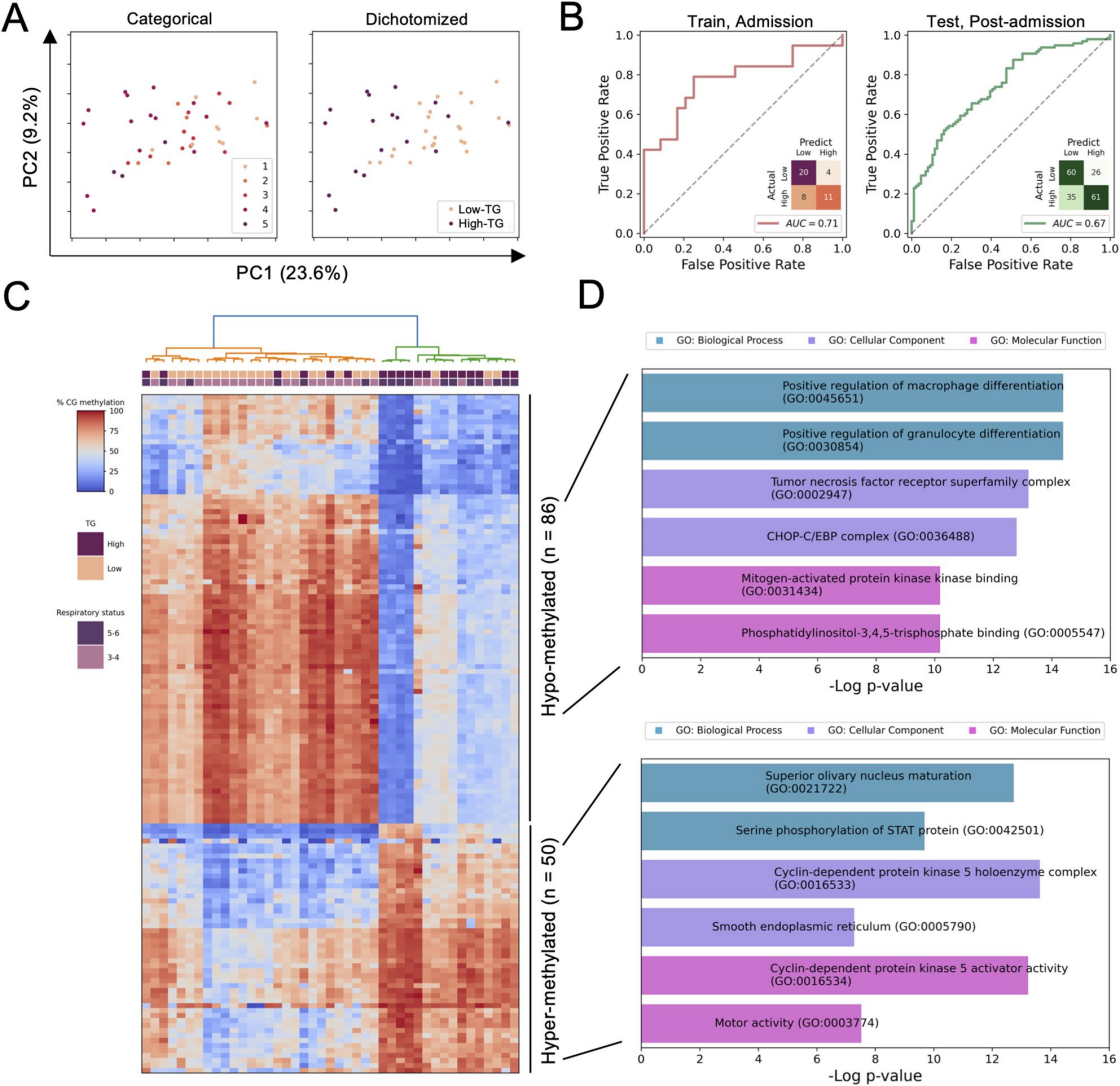
Differential DNA methylation patterns between low- and high-TG groups. **a** PCA scatter plots show that the first two PCs separate admission DNA methylomes (day 0–1, n = 43) from the rest of the cohort. **b** Logistic regression trained on admission samples informs the trajectories (low- (n = 24) and high-risk (n = 19)) of samples collected after hospitalization. **c** A total of 86 hyper-methylated and 50 hypo-methylated CpGs were identified in the high-TG group. **d** Gene Ontology (GO) enrichment analysis of the CpGs identified in **c**

The IMPACC study characterizes each patient’s TG up to 28 days after hospitalization. We asked whether it is possible to determine disease trajectory at admission based on patient’s DNA methylomes. Principal component analysis (PCA) of the admission DNA methylomes (n = 43) shows a clear separation of TG on the PC1 axis (23.6% variance, Fig. 3a), suggesting an association between DNA methylation and TG, which is less evident when longitudinal samples are included (Fig. 1b and Additional file 2: Fig. S2). We trained a penalized logistic regression model using the admission samples (day 1–2) and estimated the TG of the samples collected after (Fig. 3b). During cross validation, we exclude a sample from the training set if it is from the same patient as the test sample. Even though the training set size is small (n <= 43), the model could estimate TG with moderate accuracy (AUC = 0.67). To evaluate this finding, we further expand the “admission” from 2 to 5 days post admission, train the models and estimate TG of samples collected after. The results show that while loosening the admission definition, the informative power increases (Table 2 and Additional file 2: Fig. S6). Taken together, through restricted cross-validation and alternating the training and testing datasets, our data supports the notion that DNA methylation profiles at admission, even the day of admission, are informative of a patient’s disease trajectory.

**Table 2:** Performance of admission models

Admission	Training AUC	Testing AUC
Day 1–2	0.71	0.67
Day 1–3	0.74	0.78
Day 1–4	0.74	0.80
Day 1–5	0.88	0.85

We identified differential methylated CpG’s (DMCs), 50 hyper- and 86 hypo-methylated, in the high-TG patients (Fig. 3c and Additional file 1: Tables S4&5). Gene ontology (GO) analysis shows the hypo-methylated *loci* are proximal to genes that are enriched for macrophage and granulocyte differentiation (Fig. 3d). This could be related to the emergency myelopoiesis shown in previous studies [11, 12] and our own analysis (Fig. 2 and Additional file 1: Tables S6&7). Superior olivary nucleus maturation (GO:0021722), serine phosphorylation of STAT protein (GO:0042501) and cyclin-dependent protein kinase (GO:0016533 and GO:0016534) are the top ranked terms related to the hyper-methylated *loci*.

### Differentially methylated distal enhancers define low- and high-TG

Age, sex and obesity are associated with differences in DNA methylation [19, 37–39]. The PBMC DNA methylome is composed of DNA from a mixture of immune cells. The trait-trait correlation of inpatient samples in Additional file 2: Fig. S7 shows that there is a correlation between respiratory status and TG as expected (Spearman’s R = 0.51). PCs of cell type composition have weak associations with severity (Spearman’s R = 0.20) and TG (Spearman’s R = 0.27).

**Fig. 4:**
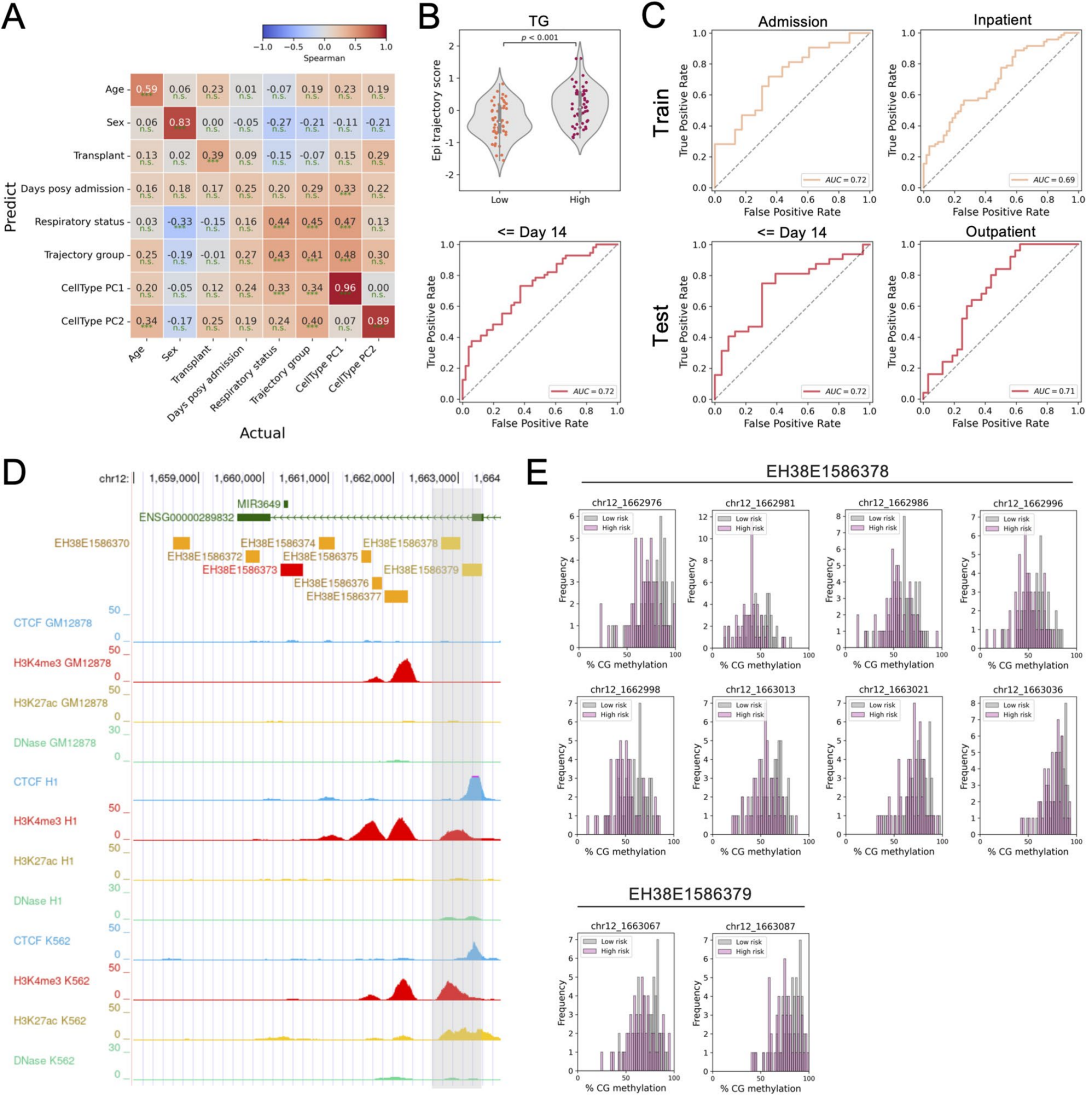
A multivariate multiple linear regression model reveals the association between DNA methylation and TG. **a** Predict-actual correlation matrix illustrating the model’s capacity to distinguish TG alongside other traits. **b** MMLR estimates (n = 182). The informed values (epi-trajectory scores) are displayed in dichotomized groups (top), and the ROC curve (bottom) illustrates the model’s ability to distinguish TG levels. **c** Cross-validation of MMLR models with different training (Admission n = 43, Inpatient n = 124) and testing data (<= Day14 n = 107, Outpatient n = 58). **d** CpG *loci* associated with TG inference in the MMLR model in **a**, **b** are located within two distal enhancers (grey) specific to the K562 cell line as identified by ENCODE. **e** Histograms show the differential methylation pattern of the 10 CpG *loci* located in the distal enhancers described in **d** between low- (n = 24) and high-TG (n = 19)

To model the contributions of these covariates to DNA methylation profiles, we constructed a multivariate multiple linear regression model (MMLR) that incorporates these key traits to explain variation in DNA methylation levels. This model can then be inverted to infer traits from DNA methylation levels (see “Methods”). Through leave-one-out-cross-validation, where all samples from a patient in the test data are excluded from the training data, the predict-actual correlation coefficients are significant for hospital stay, severity and TG (Fig. 4a and Additional file 2: Fig. S7). We termed the inferred values from the MMLR model as epi scores of each trait respectively (see “Methods”), and the epi-trajectory score served as a biomarker to classify TG (AUC = 0.72, Fig. 4b and Additional file 2: Fig. S7). To further assess model robustness, we applied longitudinal data split, training the MMLR model on inpatient samples and testing on outpatient samples, while ensuring that samples from the same patient were excluded from training to avoid overfitting (Fig. 4C), and *vice versa* (Additional file 2: Fig. S7). The model showed consistent performance (AUC = 0.71) across the training and testing sets. Together with the univariate models in Fig. 3, these results suggest that PBMC DNA methylation is associated with TG when controlling for other factors such as age, sex, obesity and cell type composition.

When testing per site association with TG in the multiple linear regression model (see “Methods”), we found 16 CpG *loci* to be significant (Figs. 4d, e and Additional file 1: Table S8). Among them, 10 CpG *loci* are captured by one probe and located within two distal enhancers, EH38E1586378 and EH38E1586379, characterized by ENCODE (Encyclopedia of DNA Elements) specifically in the K562 myelogenous leukemia cell line based on H3K27Ac deposition (Fig. 4d) [40]. Furthermore, in common myeloid progenitor cells, these enhancers are potentially accessible (Additional file 2: Fig. S7). DNA hypo-methylation is observed in high-TG across these 10 CpG *loci*, suggesting another layer of epigenetic regulation that potentially leads to an open chromatin state and drives the myelopoiesis phenotype in severe and high-TG COVID-19 patients (Fig. 4e). To be noted, we mapped these TG-specific CpG *loci* to the GoDMC mQTL database and there is no overlap [41], suggesting that the identified methylation differences are less likely to be driven by known common mQTLs and may instead reflect infection-associated epigenetic changes.

### Epigenetic memory resets after hospitalization

**Fig. 5:**
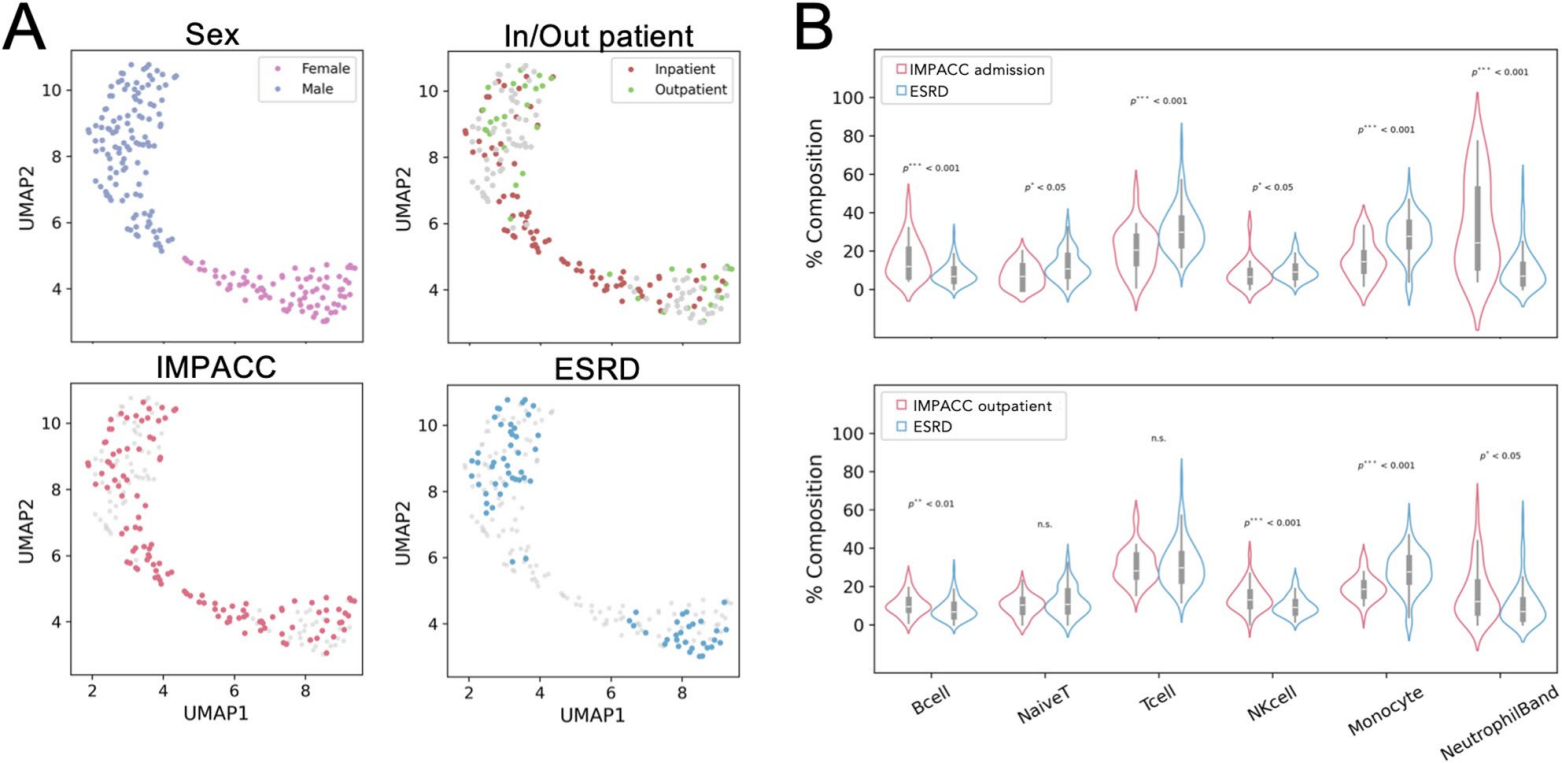
Epigenetic memory resets in outpatient cases. **a** The DNA methylation profiles of outpatient COVID-19 subjects are more similar to the ESRD COVID-negative patients (Female n = 123, Male n = 195, Inpatient n = 124, Outpatient n = 58, IMPACC n = 182, ESRD n = 136). **b** Outpatient IMPACC subjects’ cell composition (n = 58) returns to the ground state represented as ESRD (IMPACC admission n = 43, IMPACC outpatient n = 58, ESRD n = 136)

Next, we asked whether DNA methylomes return to a pre-COVID state after infection. Since IMPACC only recruited molecularly confirmed SARS-CoV-2-positive, hospitalized subjects, we compared IMPACC samples with another cohort of patients with end-stage renal disease (ESRD), the final stage of chronic kidney disease (CKD), sampled at the day of kidney transplant prior to induction medication, had neither SARS-CoV-2 infection nor COVID-19, and their PBMC DNA methylation profiles were measured using the same targeted probes [42, 43]. Figure 5A shows that the DNA methylomes grouped mainly by sex and within both sexes the IMPACC samples separate depending on whether they were hospitalized or not. Interestingly, the ESRD samples are more similar to the outpatient samples, suggesting that after hospitalization the DNA methylome differentiates toward a cleared COVID state. In the IMPACC cohort only low-TG individuals had outpatient samples, it is possible high-TG patients may maintain changes in the DNA methylome for longer. This result reflects that COVID-negative ESRD DNA methylomes vary dramatically to those from high-TG and acute infection.

We further investigated the leukocyte composition and compared IMPACC-admission and IMPACC-outpatient with the ESRD samples (Fig. 5b). The high levels of neutrophil band cells in the IMPACC-admission samples drop in the outpatient samples, and the T cell depletion is reversed back to the same level as the ESRD samples. In summary, after resolution of SARS-CoV-2 infection and COVID-19 the DNA methylome and the leukocyte composition converge to a cleared COVID state.

### Transcriptome profiles are also associated with TG

**Fig. 6:**
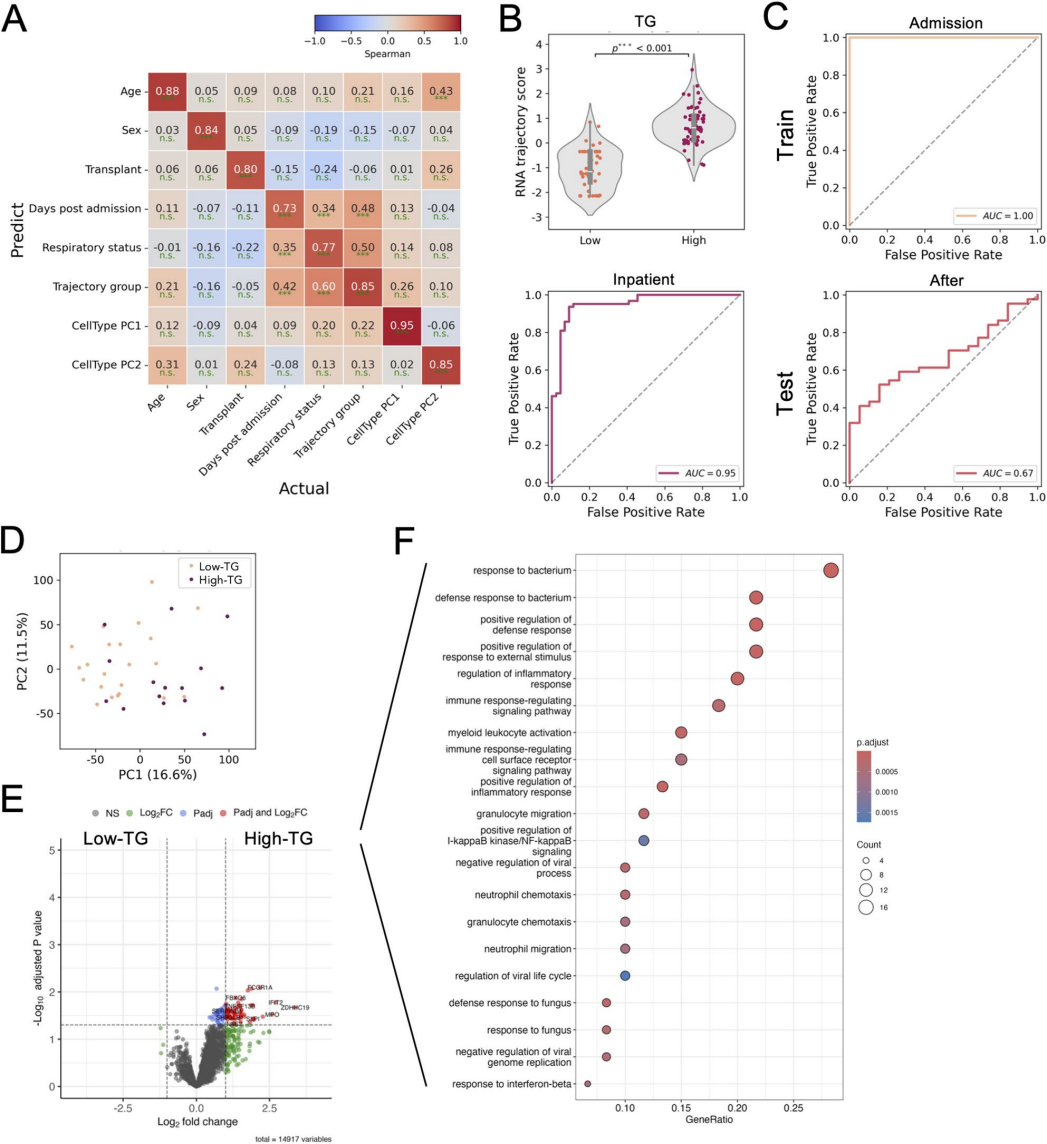
Gene expression model is temporally associated with TG. **a** The inpatient RNA-seq MMLR predict-actual correlation matrix demonstrates discriminative ability for TG as well as other traits (n = 107). **b** MMLR estimates corresponding to **a** The MMLR-informed values (RNA trajectory scores) are displayed in dichotomized groups (top, Low-TG n = 44, High-TG n = 63), and the ROC curve (bottom) illustrates the model’s ability to distinguish TG. **c** Admission MMLR model effectively distinguishes TG within the same group (top, n = 37), but its discriminative strength decreases markedly across different time points (n = 70). **d** PCA of admission transcriptomes shows separation corresponding to TG levels (admission Low-TG n = 22, High-TG n = 15). **e** Differential gene expression analysis reveals a greater number of up-regulated genes in high-TG samples, with **f** showing the related GO terms

The IMPACC study previously used PBMC transcriptomics to identify gene modules correlated with TG [2]. We therefore asked in our sub-study if the transcriptome profiles are also associated with TG. In the current single-center study we accessed the PBMC transcriptomic data from 75 participants in the UCLA cohort. The inpatient MMLR model showed a strong correspondence between predicted and actual values, with Spearman’s R = 0.85 for TG, and R > 0.70 for all other traits (Fig. 6a and Additional file 2: Fig. S8). The RNA trajectory score effectively distinguished between low- and high-TG, with an AUC = 0.95, indicating strong discriminative ability (Fig. 6b). However, when the model was testing across the hospital stay, i.e. using as longitudinal split for training and testing, its discriminative performance dropped dramatically (Fig. 6c). The admission model estimates admission samples’ TG correctly in LOOCV with AUC = 1.00 but declines to 0.67 when testing in samples collected after hospitalization (Fig. 6c). A similar performance is obtained with different training and testing sets (Additional file 2: Fig. S8). These results contrast with those obtained using DNA methylation data, where models trained on admission samples effectively generalized to post-hospitalization samples.

We next performed PCA and found that the admission transcriptomes of different TGs could be separated by PC1 and PC2 (Fig. 6d). We performed differentially gene expression analysis in the admission samples and found 65 up-regulated genes in high-TG group (Fig. 6e and Additional file 1: Table S9). Based on GO-term enrichment, these genes function mainly in defense response to bacterium, inflammatory response, and myeloid leukocyte activation (Fig. 6f), suggesting that stress-induced myelopoiesis occurs in the high-TG group.

## Discussion

Our study examines DNA methylation changes in hospitalized COVID-19 patients during the first 28 days post-admission, focusing on differences associated with TG from milder (TG1–3) to more severe disease (TG4) and death (TG5), and identifies differential DNA methylation and transcriptional patterns associated with the host response to SARS-CoV-2 infection. Given the modest sample size (n = 75), we dichotomized TG1-3 and TG4-5 samples into low- and high-TG to maintain the statistical power and to avoid overinterpretation of the results. Our results show that, on the day of admission, DNA methylation patterns in PBMCs are already associated with a patient's disease trajectory. CpG *loci* located on potential regulatory regions are also characterize. This represents the first evidence that DNA methylation may serve as a potential biomarker for forecasting COVID-19 disease progression.

Lymphopenia and emergency granulopoiesis/myelopoiesis [11, 12, 44] have been observed in severe COVID-19 and reflect the association between severity and dysregulated immune response. Manunta et al. carried out a qualitative evaluation of the density medium used to isolate PBMCs and those from COVID-19 patients are more cloudy and less transparent [45]. Their flow cytometry data shows that during acute SARS-CoV-2 infection (24 h post admission), granulocyte population shifts down on the side scatter axis [45], indicating the loss of granularity and the potential to be immature granulocytes, i.e. band neutrophils. However, to date there are no studies that have characterized band neutrophils from COVID-19 patients’ PBMC. We performed cell type deconvolution using DNA methylation signatures identified from comprehensive WGBS datasets, including the band neutrophil methylomes from BLUEPRINT [25, 26]. Our results show the longitudinal leukocyte composition dynamics in low- and high-TG and reveal that high levels of band neutrophils are present during early hospitalization and associated with patient mortality. High-TG individuals have a lower lymphoid/myeloid ratio at the day of admission and the ratio increases around day 14 to be in line with low-TG. These results suggest the presence of band neutrophils in COVID-19 PBMCs and particularly within severe COVID-19 cases, possibly caused by stress-induced myelopoiesis. In addition, we show that among COVID-19 cases that similarly require hospitalization, there is still divergence in cell type composition.

Previous studies show that male patients have higher level of pro-inflammatory innate immunity chemokines and cytokines such as IL-8 and IL-18, and there is more robust T cell response among female patients than male patients at admission [46]. However, higher levels of innate immune cytokines were associated with worse COVID progression in female patients, but not in male patients [46]. We pursued this sex-specific immune response and when stratified with sex, we found female has a lower lymphoid/myeloid ratio than male up to 2 weeks post admission. At admission, female patients show much more band neutrophils in high-TG than low-TG (p = 0.002) while male patients show no difference. We therefore proposed that female is more sensitive to COVID-related cell type composition changes, i.e. myelopoiesis, and the negative impact of increasing innate immune cytokines further lead to high-TG.

A previous study showed that the DNA methylome encodes the acute environmental exposure in mild/asymptomatic SARS-CoV-2 infection and predicts time after infection [10]. Our results support the inference of time post infection in severe COVID-19 patients who require hospitalization based on the DNA methylome. Within hospitalized patients, the DNA methylome is associated with respiratory severity. We further trained an admission model and used this to infer the follow-up clinical TGs, both the DNA methylation-only logistic regression model and the MMLR model that accounts for demographic and clinical traits generate significant associations. We also trained DNA methylation-only logistic regression model with demographic and clinical traits as covariates and generated similar results.

50 hyper- and 86 hypo-methylated CpGs were identified in the high-TG. GO-term enrichment analysis indicates the hypo-methylated *loci* are critical for macrophage and granulocyte differentiation, reflecting the myelopoiesis observed in our cell type deconvolution. We found hyper-methylation in *loci* related to CDK5R1. This gene is involved in STAT protein phosphorylation and cell cycle-related pathways, suggesting an aberrant immune response and cell growth and division. STAT proteins act downstream of interferon (IFN) mediated signaling during SARS-CoV-2 infection [3, 47] and STAT1 phosphorylation and expression increases in severe COVID-19 cases [48]. Hyper-methylation at these sites in high-TG patients at admission suggests a potential host defense mechanism through DNA methylation to silence excessive IFN production (i.e. cytokine storm) and STAT signaling pathway. After considering the major impact of leukocyte composition in the MMLR model, only CpG *loci* located in 2 distal enhancers are significantly associated with low- or high-TG. Since these 2 enhancers are specifically observed in a myelogenous leukemia cell line K562, and we also observed that in common myeloid progenitor cells these enhancers are accessible. We hypothesized they play critical roles in the stress-induced myelopoiesis in severe high-TG COVID-19 cases. Future research should perform functional assay in myelogenous cell lines to establish the molecular mechanism. Specifically, CRISPR/Cas9 knock-out or *de novo* DNA methylation/de-methylation of these enhancers and apply genomic sequencing such as single-cell RNA-seq could reveal the cell differentiation potential. Further compiling with SARS-CoV-2 infection could verify the epigenetic pathogenesis mechanism *in vitro*.

A recent study from the CHARM cohort reported that in asymptomatic or mildly symptomatic COVID-19 patients, after > 45 days the DNA methylome resets to the first day an individual tested positive [10]. We therefore investigated if severe COVID-19 patients’ DNA methylome resets during a 1-year follow-up. Through a combined analysis, we found that in later stages post-infection, after discharge from the hospital, the DNA methylome reverts back to a pseudo-ground-state which is represented herein by a COVID-negative ESRD cohort. The excessive band neutrophil fraction also reverts in the convalescent phase and is not observed in COVID-negative ESRD. These results further confirm our TBS-seq panel is able to capture COVID-19 specific immune responses and support that the stress-induced myelopoiesis/granulopoiesis being an acute-phase process and doesn’t persist after resolution of infection.

Lastly, we interrogated the association between gene transcription and TG. In contrast to DNA methylation, the gene expression MMLR models distinguish low- and high-TG well in cross-sectional analyses but demonstrate limited discriminative performance longitudinally, likely due to the transient nature of gene transcription. In addition, the 65 up-regulated genes were not correlated with either hyper- or hypo-methylated CpGs. These results suggest that in severe acute COVID-19 cases, gene expression changes dramatically across hospital stay, whereas the DNA methylation encodes the effects of TG and can be potentially used as a prognostic biomarker of TG.

Collectively, our findings reveal a layer of epigenetic regulation linked to the clinical and phenotypic progression of COVID-19 trajectories. By assessing the patients’ DNA methylation levels upon admission, we have the potential to develop biomarkers tailored to their disease trajectories. Thus, this study provides an epigenetic roadmap that is associated with longitudinal phenotypes of COVID-19.

## Conclusions

The PBMC DNA methylation capture panel and the computational algorithms presented in this study show the immune cells’ epigenomic profiles are associated with the progression of COVID-19 during the acute phase of infection. These findings provide the first evidence correlating phenotypic disease trajectories with epigenetic profiles and identify potential CpG *loci* within enhancer regions that may serve as biomarkers for identifying patients at increased risk of developing severe COVID-19.

### Limitations of the study

Our study has several limitations, including (1) The modest number of subjects (n = 75), (2) it is a single-center study subset from a multi-center cohort, (3) the lack of standard “ground state” that defines COVID-negative by a standard assay, (4) the enrollment timing excludes the impact of vaccination and was before the outbreak of specific viral variants such a B.1.617.2 (Delta) and B.1.1.529 (Omicron), (5) important *loci* could be missed from the TBS-seq panel, (6) lack of paired CyTOF from PBMC, (7) the medical treatment variations were not accounted for, (8) pregnant women and children were excluded, and (9) the average age is around 61 years old and the conclusions may not generalize for all age groups. The single-center study may limit the generalizability since the center-specific practice such as the sample collection and processing, assay protocols, clinical management and patient recruitment procedures usually vary across centers. Patient-wise, age, sex, ethnicity, socioeconomic status, lifestyle factors and comorbidities might differ by center or its geographic location. While the IMPACC study benefits from broad enrollment that reflects real-world incidence, this also introduces variability and a lack of control. The primary outcome TG is a composite variable derived from multiple internal parameters specific to the IMPACC cohort, and comparable publicly available datasets with equivalent outcome definitions are limited for validation. Additionally, the ESRD cohort, used as a pseudo-ground state in this study, is limited as it consists of patients with COVID-negative end-stage kidney disease rather than healthy controls. Future research applying similar assays to other subsets of the IMPACC cohort could help strengthen these conclusions. This TBS-seq approach together with the epi-trajectory score derived per patient could be established as a new modality to characterize patients at high risk of developing severe outcomes. In addition, the DNA methylome dynamics in long COVID could be investigated accordingly.

## Supplementary Information

Below is the link to the electronic supplementary material.

Supplementary Material 1

Supplementary Material 2

## Data Availability

TBS-seq data has been deposited to Gene Expression Omnibus (GEO) the under the accession number GSE298460 (https://www.ncbi.nlm.nih.gov/geo/query/acc.cgi?acc=GSE298460). The analysis pipeline has been deposited to https://gitlab.com/fmhsu0114/impaccmethylation.

## Abbreviations

COVID-19: Coronavirus disease 2019
IMPACC: Immunophenotyping Assessment in a COVID-19 Cohort
TBS-seq: Targeted bisulfite sequencing
ESRD: End-stage renal disease
DMR: Differentially methylated regions
LOOCV: Leave-one-out cross validation
CyTOF: Cytometry by time of flight
