# Supplementary material for: DNA methylation profiles at hospital admission are associated with subsequent severe COVID-19 outcomes
Source: Clin Epigenetics. 2026 Apr 22;18:112. doi: 10.1186/s13148-026-02138-5 (PMC13251264; doi:10.1186/s13148-026-02138-5)
Supplement: Supplementary file 2 — Supplementary Material 2 [file 13148_2026_2138_MOESM2_ESM.pdf]

DNA methylation profiles at hospital admission are associated with subsequence severe COVID-19 outcomes

Additional file 2: Figure S1-S8

Fei-Man Hsu[1,2], Harry Pickering[3], Steve E. Bosinger[4], Walter Eckalbar[5], Holden T. Maecker[6], Seunghee Kim-schulze[7], Al Ozonoff[8], Joann Diray-Arce[8], Joanna M. Schaenman[9], Elaine F. Reed[3,*], and Matteo Pellegrini[2,*]

[1] Smart Medicine and Health Informatics Program, International College, National Taiwan University, Taipei, Taiwan

[2] Department of Molecular, Cell and Developmental Biology, University of California Los Angeles, Los Angeles, CA, USA

[3] Department of Pathology and Laboratory Medicine, David Geffen School of Medicine, University of California Los Angeles, Los Angeles, CA, USA

[4] Emory School of Medicine, Atlanta, GA, USA

[5] Department of Medicine, University of California San Francisco, San Francisco, CA, USA

[6] Stanford University School of Medicine, Palo Alto, CA, USA

[7] Icahn School of Medicine at Mount Sinai, New York, NY, USA

[8] Clinical and Data Coordinating Center, Boston Children's Hospital, Boston, MA, USA

[9] Department of Medicine, David Geffen School of Medicine, University of California Los Angeles, Los Angeles, CA, USA

[*] Senior author, corresponding author

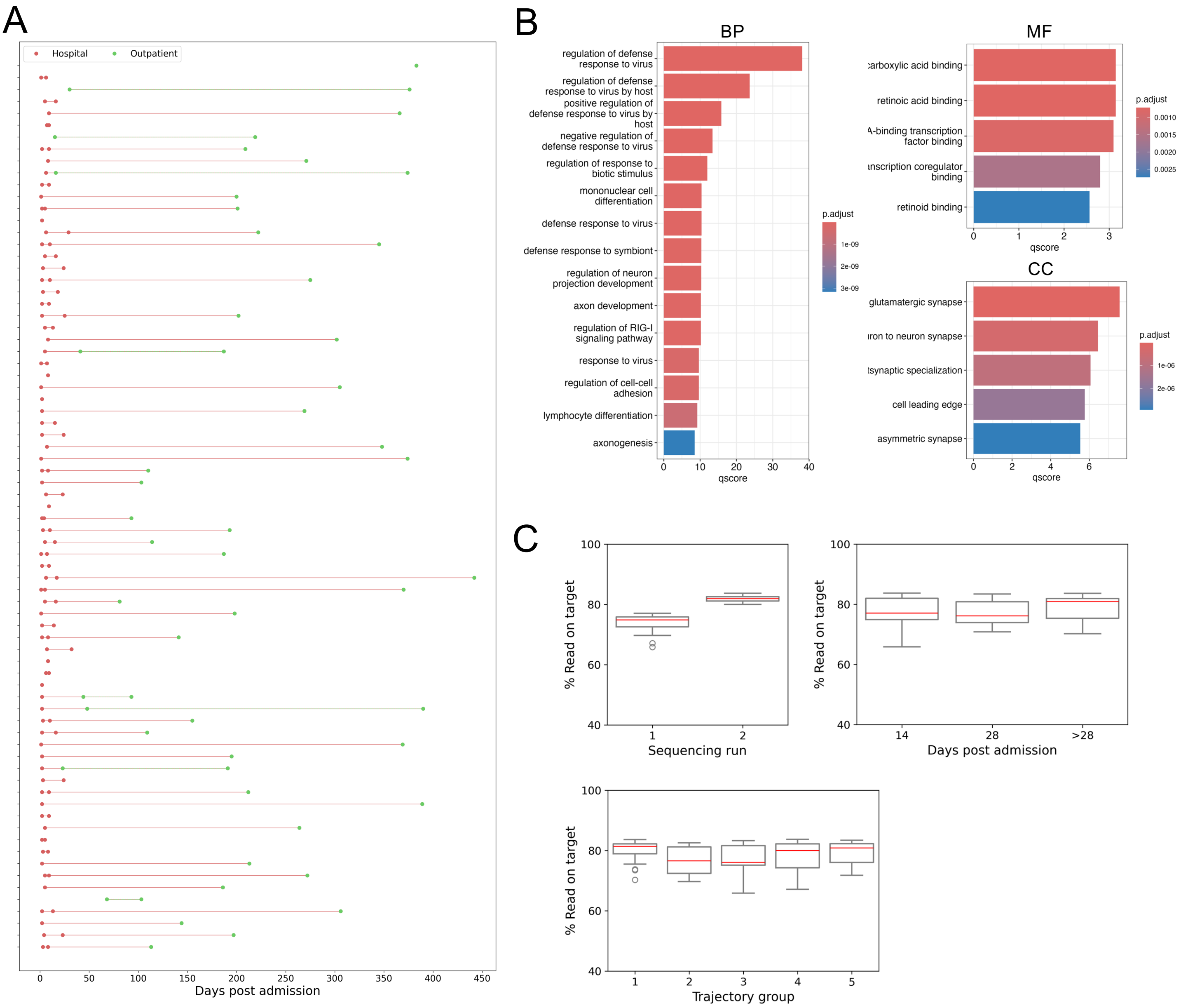

Figure S1 Overview of TBS-seq samples, related to Figure 1.

(A) Sample collection overview

(B) Gene ontology (GO) of genome regions in this TBS-seq probe panel

(C) QC of the 2 sequencing runs

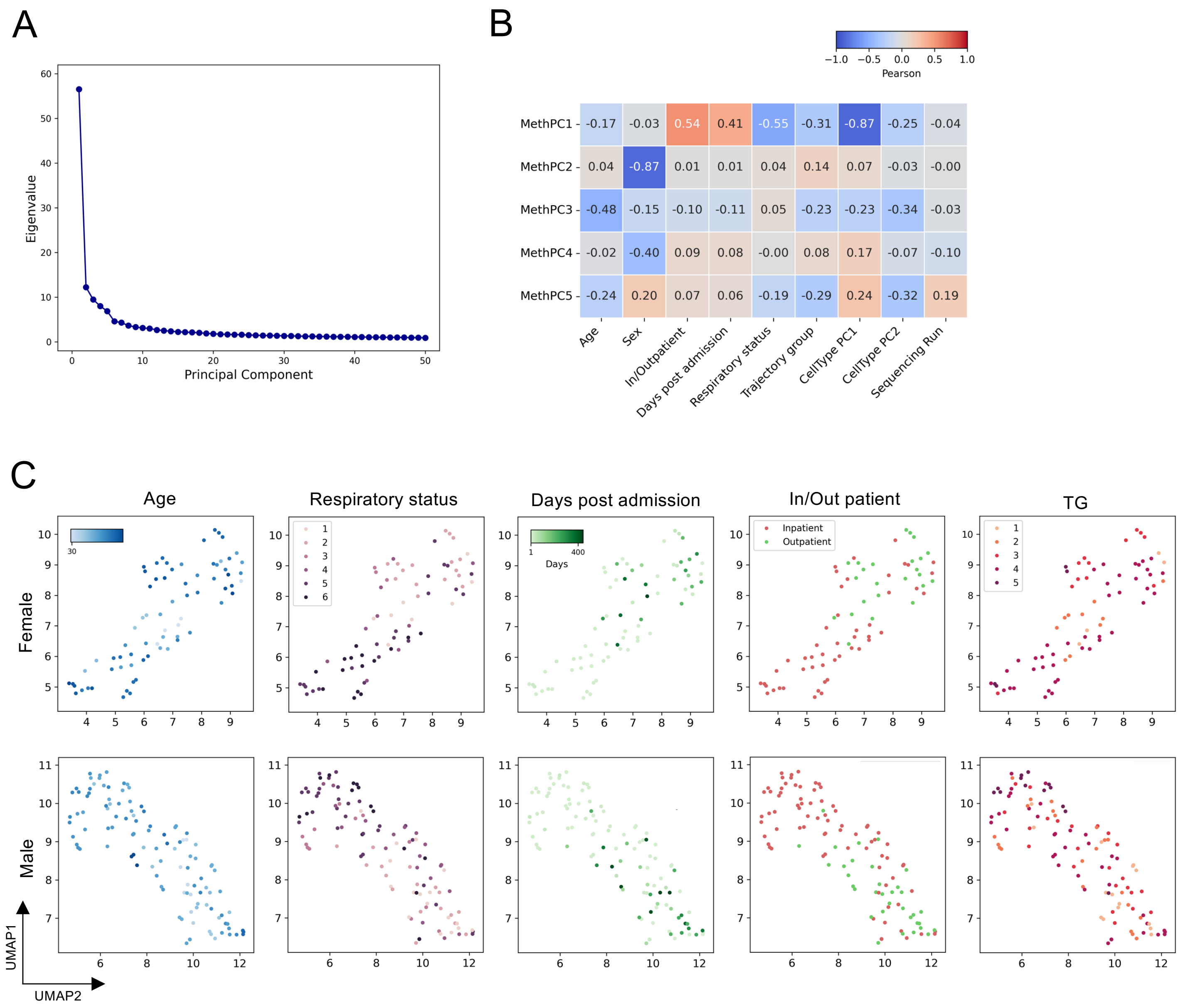

Figure S2 Dimensional reduction of DNA methylome, related to Figure 1.

(A) Scree plot of methylation PCs

(B) PCA correlation matrix

(C) Sex-stratified UMAPs

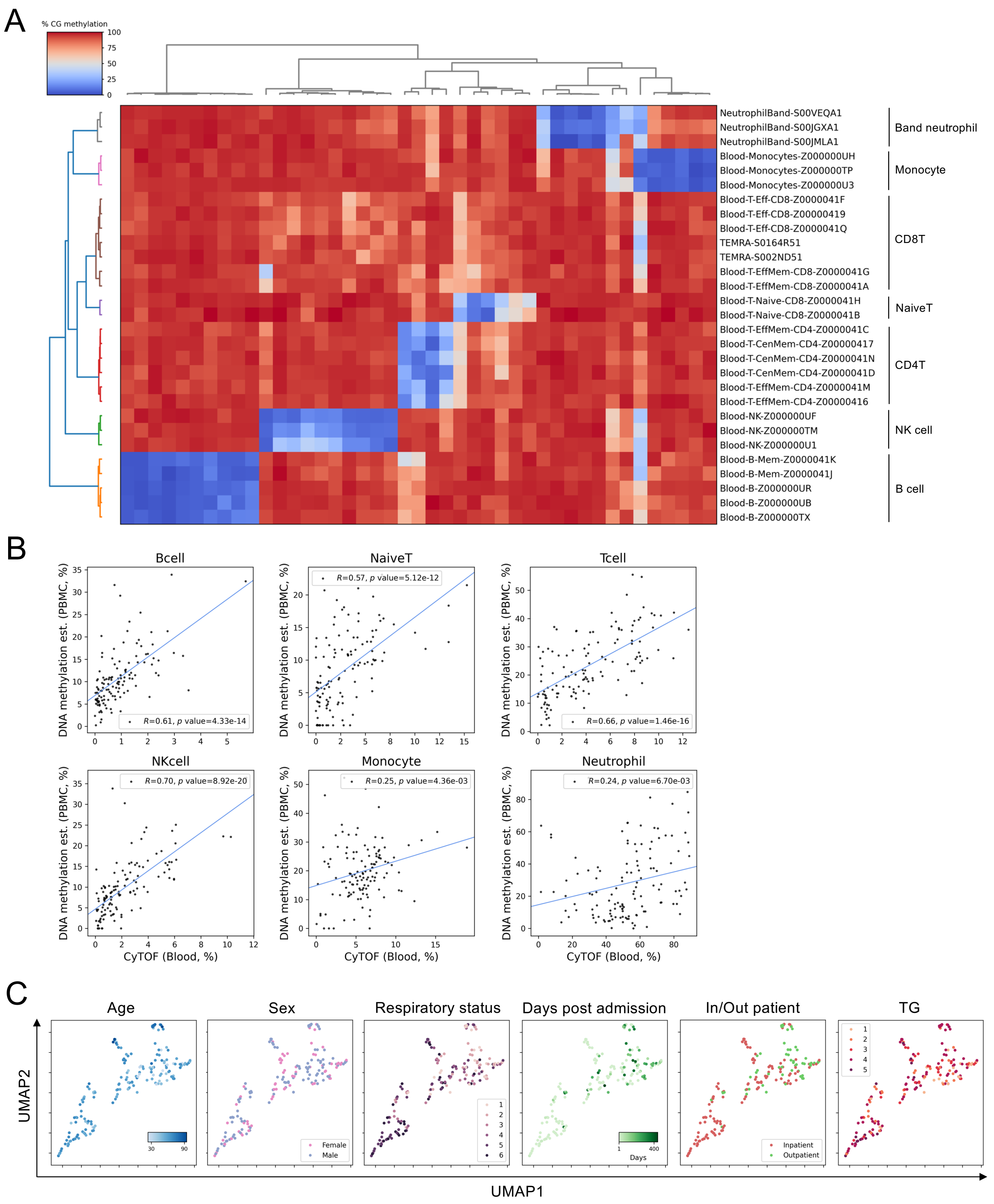

Figure S3 TBS-seq captures immune cells' epigenetic signatures to deconvolute blood cell types, related to Figure 2.

(A) DMRs used to deconvolute PBMC

(B) Scatter plots of cell type composition estimated by DNA methylation and CyTOF

(C) DNA methylome corrected for cell types visualized with traits in UMAP coordinates

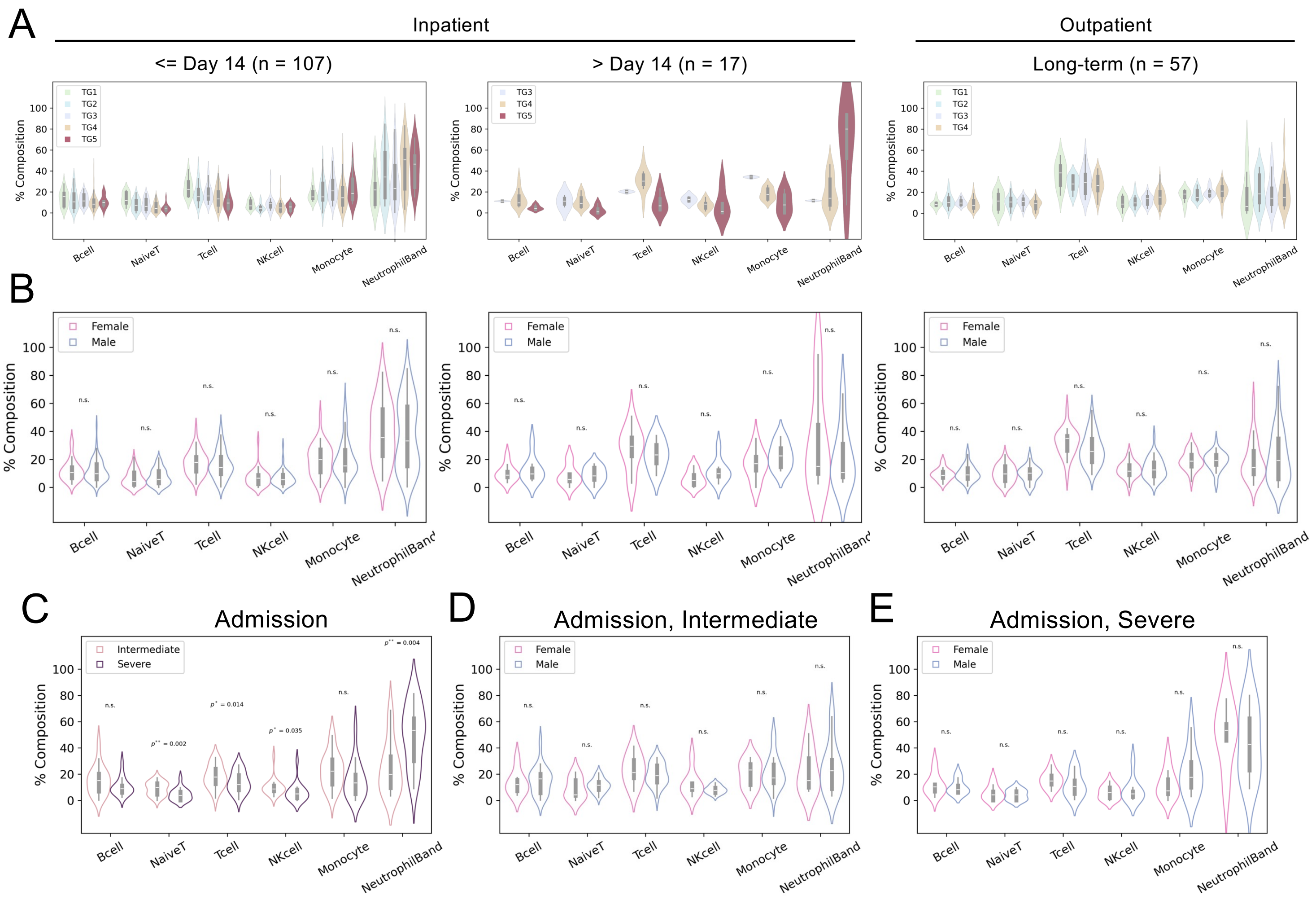

Figure S4 Leukocyte composition stratified with traits, related to Figure 2.

(A) Inpatient samples stratified by TG

(B) Inpatient samples stratified by sex

(C) Admission samples stratified by respiratory status

(D) Admission samples with intermediate respiratory status stratified by sex

(E) Admission samples with severe respiratory status stratified by sex

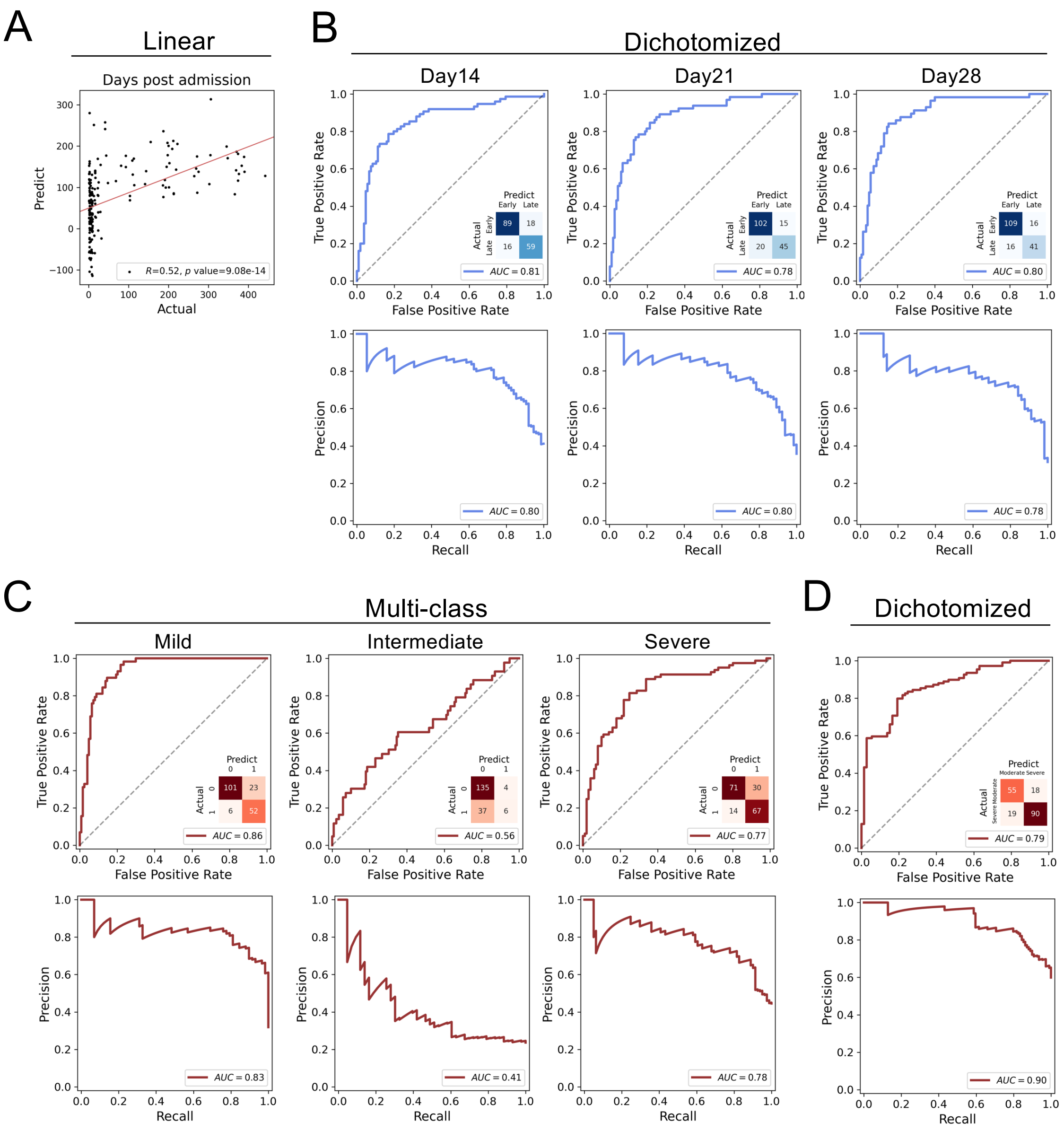

Figure S5 Supporting machine learning models, related to Figure 3.

(A) Predict-actual correlation of days post admission

(B) Performance of models predicting dichotomized days post admission

(C) Multi-class model to predict respiratory status

(D) Logistic regression model to predict dichotomized respiratory status

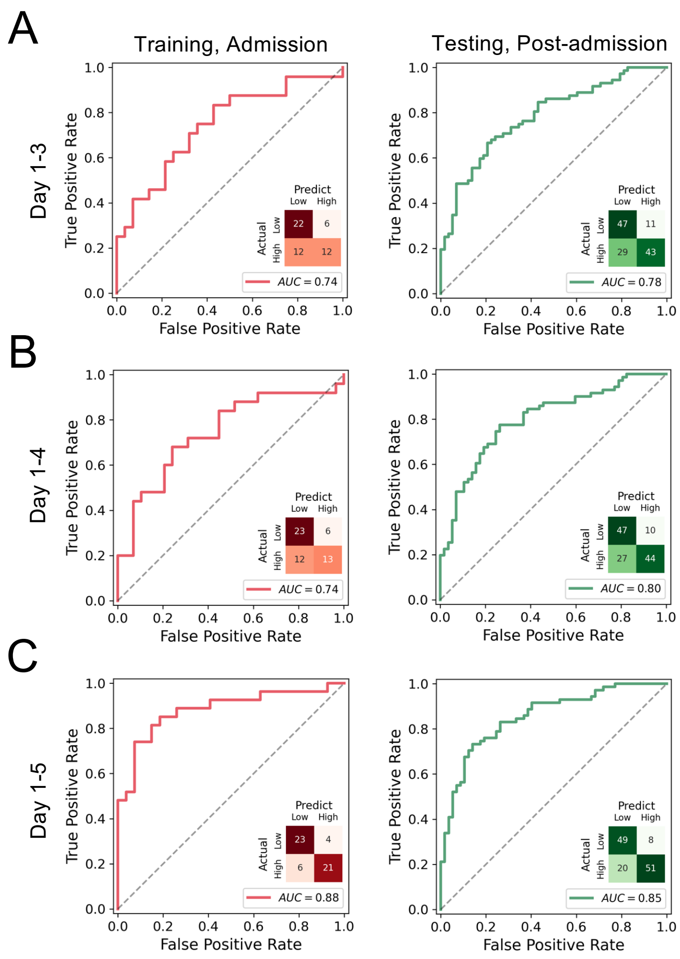

Figure S6 Supporting baseline models, related to Figure 3.

Model performances using (A) day 1-3, (B) day 1-4 and (C) day 1-5 as admission training data (left) and predicting the TG in samples collected after (right).

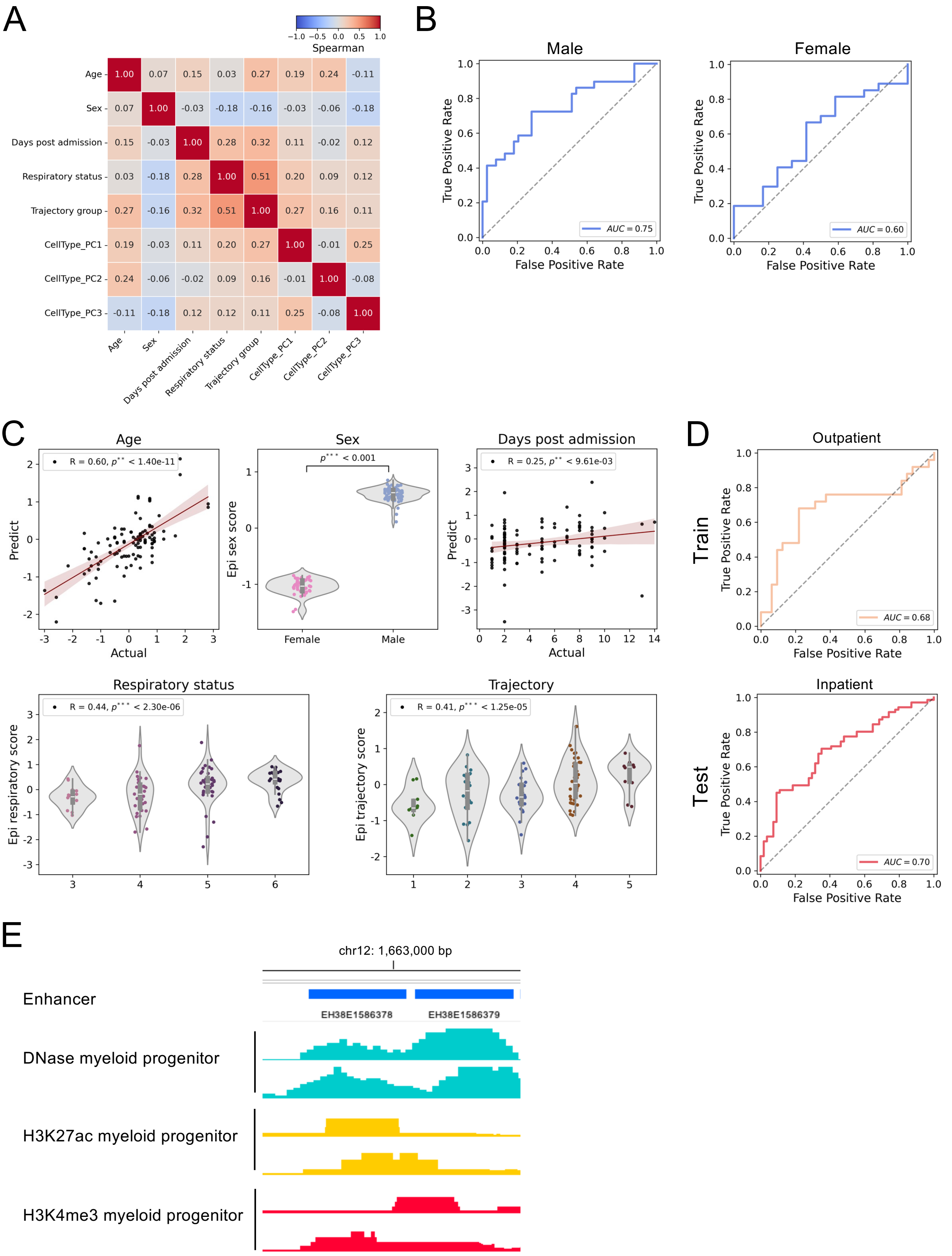

Figure S7 Supporting data for the MMLR model, related to Figure 4.

(A) Trait-trait correlation matrix

(B) Sex-stratified MMLR model performance

(C) Predict-actual correlation of each trait

(D) Performance of MMLR model trained with Outpatient and test in Inpatient

(E) ENCODE distal enhancers are active in $CD34^{+}$ myeloid progenitor cells

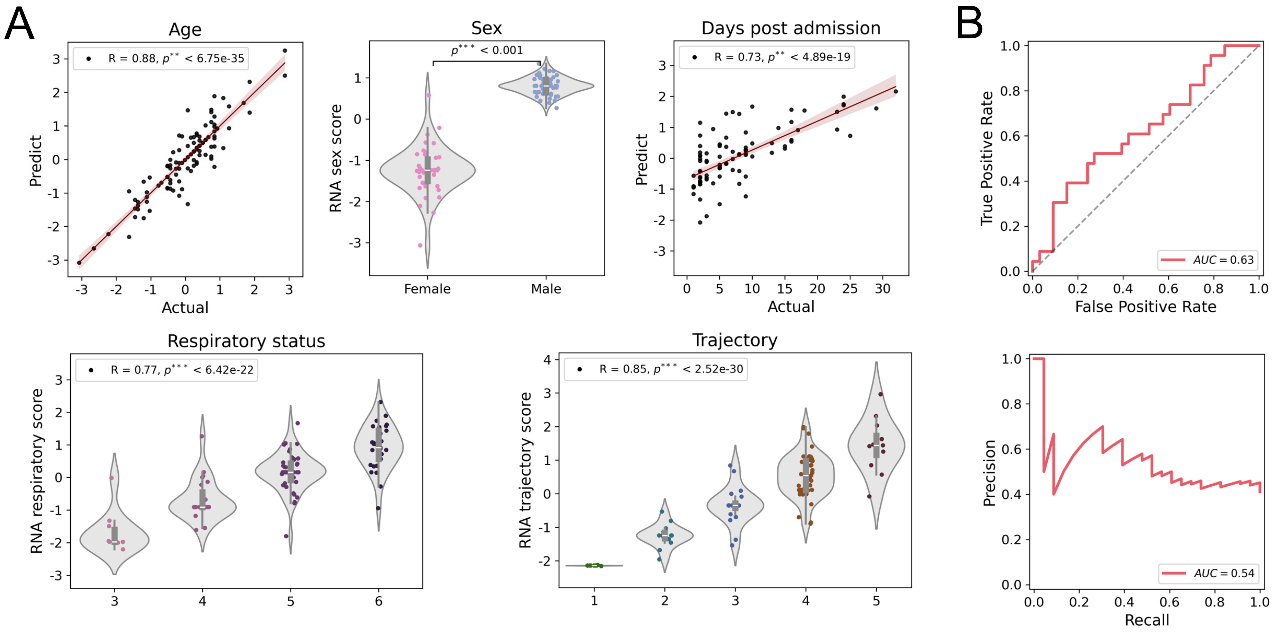

Figure S8 Supporting data for the transcriptome MMLR model, related to Figure 6.

(A) The predict-actual correlation of each trait in the MMLR model.

(B) ROC and PRC of MMLR model trained with Inpatient and test in outpatient.
